# Periodic Prompts and Reminders in Health Promotion and Health Behavior Interventions: Systematic Review

**DOI:** 10.2196/jmir.1138

**Published:** 2009-05-14

**Authors:** Jillian P Fry, Roni A Neff

**Affiliations:** ^2^Health Policy and Management DepartmentJohns Hopkins Bloomberg School of Public HealthBaltimoreMDUSA; ^1^Center for a Livable FutureJohns Hopkins Bloomberg School of Public HealthBaltimoreMDUSA

**Keywords:** Prompt, limited contact intervention, eHealth, behavior change

## Abstract

**Background:**

Health behavior interventions using periodic prompts have utilized technology, such as the Internet, that allows messages to be sent to participants in cost-effective ways. To our knowledge, no comprehensive evidence review has been performed specifically to evaluate the effectiveness of communicating regular messages and to examine how characteristics of the prompts change the effectiveness of programs aimed at reminding people to adopt healthy behaviors, maintain those they already practice, and cease unhealthy behaviors.

**Objective:**

A systematic literature review was performed to investigate the effectiveness of limited contact interventions targeting weight loss, physical activity, and/or diet that provided periodic prompts regarding behavior change for health promotion. The review sought to identify specific characteristics of these interventions that may be associated with superior results.

**Methods:**

Electronic literature searches were performed between February and April, 2008. Articles were included if periodic prompts were used as an intervention or a component of an intervention, a behavioral or biological outcome measure was used, and an ongoing health promotion behavior was targeted. A rating system was applied to each study to provide a quantitative representation of the quality of the evidence provided by each article.

**Results:**

There were 19 articles with a combined sample size of 15,655 that met the inclusion criteria, and 11 studies reported positive findings regarding the utility of periodic prompts. Several articles showed enhanced effectiveness when prompts were frequent and personal contact with a counselor was included. Long-term behavior change and health improvements were not examined by this review because of a lack of long-term follow-up in the literature.

**Conclusions:**

In light of promising results of most studies, additional research on limited contact interventions targeting health behaviors including weight loss, physical activity, and/or diet is merited that utilizes rigorous methods including control groups; follow-up data collection; and testing of prompt frequencies, specific intervention components, or prompt characteristics. Future research would be especially valuable if it improves understanding of the most effective types of periodic prompts for fostering long-term behavior change in order to maximize use of this tool in limited contact health promotion programs. Specifically, various types of communication technology should be used and evaluated to expand and refine their use.

## Introduction

Periodic prompts that encourage healthy behaviors are a way to remind and help motivate people to change their health behaviors. We define periodic prompts as messages, reminders, or brief feedback communicated to participants multiple times over the duration of an intervention. Prompts can be delivered at various intervals such as daily, weekly, or monthly, and can be sent using email, telephone, and mail. Health promotion professionals use periodic prompts both as standalone interventions and also as components of interventions [1]. Some interventions use personalization and tailoring in an attempt to increase prompts’ relevance to recipients [2]. Some programs also utilize counselors to communicate periodic messages, although this type of intervention requires more resources than interventions that are automated [3]. Although it might make intuitive sense to some that it is valuable to communicate regular messages to remind people to initiate or maintain healthy behaviors, to our knowledge no comprehensive evidence review has been done specifically to evaluate the effectiveness of using periodic prompts and examine which characteristics of prompts work best in health promotion interventions. A review article published in 2001 examined interventions that used one or more computer-generated messages aimed at increasing medication adherence or immunization uptake and improving chronic disease management, as well as other health behaviors [4]. This review builds upon that work by examining interventions targeting other health behaviors, evaluating the effect of periodic health prompts within health promotion interventions, and updating the literature.

A systematic literature review was performed to investigate the effectiveness of periodic prompts regarding behavior change and to identify specific characteristics of prompts that may be associated with superior results. The literature review was feasible because of the increasing use of limited contact interventions due to widespread access to the Internet and other media that are used to communicate prompts. Data from a Pew Internet and American Life Project survey in May 2008 revealed that 73% of US adults go online, and 78% have cellular phones [5]. The potential impact that increased access to technology can have on an individual’s health is great, and many organizations are recognizing the potential value of eHealth, which refers to “health services and information delivered or enhanced through the Internet and related technologies” [6]. Finding ways to implement behavior change interventions with large audiences in cost-effective ways is important due to the overwhelming challenges facing public health agencies and the limited resources available to meet them.

Health promotion studies using emerging technology are becoming more common, and researchers are tasked with balancing cost and personalization, and measuring the effects those two competing characteristics have on effectiveness of interventions. E-Health is a rapidly growing field [7,8] with advantages regarding cost and reach, and it is our hope that this review will serve to inform program development of eHealth interventions using periodic prompt messaging.

## Methods

To identify peer-reviewed articles examining the use of periodic prompts for health promotion interventions, electronic literature searches were performed between February and April of 2008. Databases and search tools accessed, with the number of articles found in initial searching, included: PubMed (1119), PsycINFO (394), Google Scholar (142), CINAHL (148), and Web of Science (444). No publication date parameter was used to exclude older articles, and all searches included articles published up to the date of the search. Searches utilized the following terms in various combinations: prompt, weekly, reminder, email, Internet, Web-based, limited contact, intervention, health, and promotion. In addition, references of articles that were identified through searching were reviewed. Twenty-four additional articles were identified through this process.

Articles were included if periodic prompts were used as a standalone intervention or were part of a larger program; a biological or behavioral outcome measure was used; and ongoing health promotion behaviors such as weight loss, physical activity, and diet were targeted. Studies aiming to change compliance with immunization or health screening guidelines were not included because of the intermittent nature of those activities. After reviewing titles and abstracts to identify relevant articles, reviewing references to locate additional articles, and applying the inclusion criteria, 19 articles were included. A meta-analysis was not feasible due to the varying data collection methods and outcome measures.

A rating system was used to represent quantitatively the quality of the evidence provided by each article included in the review. The rating system was adapted from a review article that examined studies that used one or more computer-generated patient contacts aimed at increasing medication adherence or immunization uptake and improving chronic disease management, as well as other health behaviors [4]. The authors of that review created the rating based on recommendations from the literature [9,10]. The rating system is described in Table 1, which is a modified version of a table in the review article [4]. Articles were rated by the lead author, and the range of possible scores is 0 to 10. No minimum score was used to exclude studies from the review.

**Table 1:** Rating system

Factor	Description	Possible Points
Randomization	Assignment to different interventions by chance	2
Control Group	Comparison made to group of subjects not given the health behavior intervention	2
Sampling	Sampling method described Sample composition clearly described Sample of adequate size Number and ratio of withdrawals described	3
Analysis of Main Effect Variables	Clear definitions for each variable Clear description of methods and results Numeric table presented for each effect variable	1
Follow-up	Follow-up data collection to measure effects beyond immediate findings	1
Content	Intervention clearly described and replicable Discussion of withdrawals Discussion of study limitations	1

## Results

This section describes the interventions, study designs, and main findings. The main findings are broken down into the following sections: prompt frequency, medium used, intervention components, tailoring, and level of interaction with intervention. Table 2 and Table 3 present descriptive information, main findings, and a quality score for each article.

### Descriptive Findings

The 19 articles were published between 1988 and 2008, with 17 of the studies published after the year 2000. Study sample sizes ranged from 43 to 7743, with a median sample size of 190 participants. There were a total of 15,655 participants in all 19 of the studies described in the journal articles, of which 12,697 (81%) participated in the four largest studies. These four studies had sample sizes ranging from 1032 to 7743 and an average sample size of 3174. Approximately 65% of all the subjects were women. While the largest study had 60% women, 14 of the 19 articles had over 70% women, including 4 that included only women.

Randomized controlled trials made up 13 of the 19 studies (articles 2, 3, 5, 6, 7, 8, 10, 12, 14, 15, 16, 17, 18 in Table 2 and Table 3 [hereafter, article numbers refer to Table 2 and Table 3]). Six conducted follow-up data collection between 3 and 12 months post-intervention (articles 6, 8, 11, 13, 17, 18). Two studies used randomization for group assignment but did not include control groups (articles 3 and 9). Four studies were observational with only one group in each study (articles 1, 4, 11, 19). The range of the quality score scale is 0 to 10, and assigned scores for the 19 articles in this review range in value from 4 to 9. The four studies with scores of 4 or 5 (articles 1, 4, 11, and 19 in Table 2 and Table 3) did not include a control group, and therefore no randomization could take place (reducing the total possible points to 6). These studies are less informative because of their study designs, but they are included in this review because the interventions used periodic prompts and outcome measures were included.

Eight of the interventions aimed to increase physical activity, seven focused on weight loss as the outcome, one was aimed at weight loss maintenance, two sought to improve dietary habits, and one focused on both physical activity and nutrition.

In terms of periodicity, 12 out of 19 studies sent prompts every week, and two studies sent prompts every 2 weeks. One program sent 5 prompts over 8 weeks, and one sent them every 5 weeks. One study sent prompts at variable time periods, allowing participants to pace themselves and providing prompts as lessons were completed. Two interventions compared weekly prompts to prompts sent less frequently (eg, every 3 weeks and monthly).

The type of periodic prompts, and the ways they were integrated with other intervention components, varied. The following are methods utilized in the most studies. In terms of mode of prompt administration, 13 interventions sent only email prompts to participants, and two studies used only telephone prompts. Seven studies used only online tools in addition to prompts, and three articles used pedometers and step logs. Two studies used in-person sessions and online tools along with prompts. Some type of tailoring was used in 14 of the 19 studies to provide personalized information to participants as part of periodic prompts. Contact with a counselor was used in 9 articles. Six studies reported on associations between the level of interaction participants had with the intervention and outcomes. Level of interaction with an intervention was measured as weeks a participant took part in the intervention, number of log ins to the intervention website, or amount of use of the online tools on an intervention website.

Intervention length ranged from 6 weeks to 30 months. The median and mode intervention time-span was 3 months (or 12 weeks), with five studies implementing interventions of this duration.

**Table 2:** Study characteristics

	Article	N	Health Behavior	Intervention Duration	Study Design	Control Group	Follow-up	Additional Intervention Components
1	Block 2004 [11]	84	nutrition	12 weeks	observational, single group, pretest-posttest; set up to test effect of weekly emails and online tools in moving people forward in stage of change, decreasing fat intake, and increasing fruit and vegetable consumption	no	no	online tools: goal setting and bulletin board
2	Conn 2003 [12]	190	physical activity	3 months	randomized, four groups (2 x 2 design), pretest-posttest; set up to test effect of motivational interviewing and weekly prompts aiming to increase exercise; groups: (1) motivational interviewing and prompts, (2) motivational interviewing only, (3) prompts only, (4) control	yes	no	motivational interviewing
3	Dinger 2007 [13]	74	physical activity	6 weeks	randomized, two groups, pretest-posttest; set up to test effect of e-mails based on the transtheoretical model (TTM) on walking; second group wore pedometers, submitted step logs, and received weekly reminder emails, first group also received emails based on the TTM	no	no	pedometers and step logs
4	Dinger 2005 [14]	43	physical activity	6 weeks	observational, one group, pretest-posttest; set up to test effect of intervention including pedometers, a brochure, and emails targeting TTM constructs on walking behavior and changes in TTM constructs	no	no	pedometers, step logs and brochures
5	Hunter 2008 [15]	446	weight	6 months	randomized, two groups, pretest-posttest; set up to test effect of a behavioral Internet intervention using online tools, two brief motivational interviewing phone calls, and personalized feedback compared to usual care	yes	no	online tools: self-monitoring tools for food and exercise, weight tracking chart, weekly lessons; two brief motivational interviewing phone calls
6	Jeffery 2003 [16]	1801	weight	self-paced	randomized, three groups, pretest & two posttests; set up to test effect of an interactive 10-lesson intervention on weight loss where feedback was delivered by mail or telephone, compared to usual care	yes	yes	ten paper-and-pencil lessons
7	King 1988 [17]	52	physical activity	6 months	randomized, two groups, pretest-posttest; set up to test effect of periodic phone calls on amount of exercise and level of oxygen consumption	yes	no	baseline exercise instruction session
8	Lombard 1995 [18]	135	physical activity	12 weeks	randomized, five groups (2 x 2 plus a control group), repeated measures; set up to test effect of prompting frequency (weekly versus every 3 weeks) and prompt structure (high versus low); five groups: (1) weekly prompts with high structure, (2) less frequent prompts with high structure, (3) weekly prompts with low structure, (4) less frequent prompts with low structure, (5) no prompts	yes	yes	walking logs and instruction on how to start walking groups
9	Marshall 2003 [19]	655	physical activity	8 weeks	randomized, two groups, pretest-posttest; set up to test effect of (1) booklet with reinforcement letters and (2) a website with reinforcement emails to affect stage of change and increase physical activity	no	no	booklet or online tools: quizzes, goal setting, activity planning, and target heart rate guide
10	Napolitano 2003 [20]	65	physical activity	12 weeks	randomized, two groups, pretest & two posttests; set up to test effect of an intervention website based on the social cognitive theory with weekly emails on moving people forward in stage of change and increasing physical activity	yes	no	website based on the Social Cognitive Theory
11	Petersen 2008 [21]	7743	weight	self-paced	observational, one group, pretest & two posttests; set up to test effect of a multi-component online intervention on changing stage of change, dietary habits, exercise, and weight	no	yes	online tools: food and weight tracking tools, progress reports, weekly newsletters, community support, expert assistance, “SparkPoints”
12	Plotnikoff 2005 [22]	2121	physical activity & nutrition	12 weeks	randomized, two groups, pretest-posttest; set up to test effect of email messages based on the social cognitive theory on physical activity, dietary changes, and social cognitive theory constructs	yes	no	weekly prompts only
13	Spittaels 2007 [23]	379	physical activity	8 weeks	randomized, three groups, pretest-posttest; set up to compare the effect of (1) computer-tailored online advice with 5 emails based on the stage of change theory, (2) tailored online advice with no emails, and (3) online advice only	no	yes	online physical activity advice
14	Svetkey 2008 [24]	1032	weight loss maintenance	30 months	randomized, three groups, pretest & five posttests; set up to compare effect of three conditions on weight loss maintenance: (1) monthly personal contact, (2) interactive technology-based intervention, (3) self-directed control group	yes	no	technology-based intervention: online tools- social support, self-monitoring, check-in accountability, problem solving and relapse prevention training; personal contact: met or spoke with interventionist monthly; control: pamphlet and one brief meeting with interventionist
15	Tate 2006 [25]	192	weight	6 months	randomized, three groups, pretest & two posttests; set up to compare effect of free weight loss website with no counseling to two counseling groups who had access to a more comprehensive weight loss website; the two counseling groups were (1) automated counseling and (2) feedback from a weight loss counselor	yes	no	online tools: weekly reporting and graphs, tips, recipes, e-buddy system, diary, message board, and behavioral lessons
16	Tate 2003 [26]	92	weight	12 months	randomized, two groups, pretest-posttest; set up to compare effect of weight loss programs: (1) Internet only (2) Internet plus behavioral e-counseling (regular email communication with a counselor)	yes	no	one in-person counseling session, weekly weight, calorie and exercise reporting
17	Tate 2001 [27]	91	weight	3 months	randomized, two groups, pretest & two posttests; set up to compare effect of weight loss programs: internet education (had access to website with weight loss links) and Internet behavioral therapy (access to website plus weekly lessons, online diaries, bulletin board, and individualized therapist feedback)	yes	yes	one in-person group session; online tools: weight loss links, weekly lessons, online submission of self-monitoring diaries, and a bulletin board
18	Williamson 2006 [28]	80	weight	12 months	randomized, two groups, pretest & four posttests; set up to compare the effect of a passive health education program (a few educational sessions and access to an informational website) and an interactive behavior therapy program (nutrition education and internet counseling)	yes	yes	four in-person sessions, weekly lessons with quizzes, regular email communication with counselor, weight and activity graphs, and food intake monitoring tool
19	Woodall 2007 [29]	380	nutrition	4 months	observational, one group, pretest-posttest; set up to determine effect of an intervention consisting of an informational website and reminder emails alerting participants of new content on website	no	no	online tools: health benefit information, recipes, community directory, links related to fruit and vegetable intake

**Table 3:** Prompt characteristics, research questions, and findings

		Prompt Characteristics			Findings		
	Article	Prompt Frequency	Medium Used for Prompt	Tailoring	Level of Interaction	Summary of Results	Score (on a scale of 0 to 10)
1	Block 2004 [11]	weekly	email	yes, by lifestyle factors and chosen dietary behavior	positive association found between number of weeks a participant interacted and stage of change	• responders not in action or maintenance stages at baseline: 65% progressed for fat reduction and 74% progressed in stage for fruit and vegetable consumption • weeks a participant interacted with program related to change in stage • decrease in dietary fat and increase in fruits and vegetables observed	5
2	Conn 2003 [12]	weekly	telephone and mail	none	n/a	• prompted participants significantly increased their exercise compared to those not prompted • motivational interviewing did not have a significant effect on amount of exercise	9
3	Dinger 2007 [13]	weekly	email	yes, by stage of change for intervention group (second group received non-tailored reminders)	n/a	• both groups significantly increased amount of walking • no difference between groups' amount of walking or stage movement.	6
4	Dinger 2005 [14]	weekly	email	none	n/a	• total walking minutes significantly increased • six of the TTM constructs measured improved, but self-efficacy was not effected	4
5	Hunter 2008 [15]	weekly	email	yes, a counselor provided weekly feedback by email	positive association found between use of intervention website and weight loss	• intervention group lost more weight than usual care group, had significant BMI reduction, percent body fat reduction, and waist circumference reduction • more weight loss was associated with more use of intervention website	9
6	Jeffery 2003 [16]	varied (self-paced)	telephone or mail	yes, personalized feedback from counselor by mail or telephone	n/a	• telephone group lost significantly more weight than usual care group at 6 months • 12 month differences were not significant for either treatment group • more agreed to take part in the intervention if in the mail group • higher percentage in phone group completed all lessons	9
7	King 1988 [17]	every 2 weeks	telephone	yes, counselor provided further instruction and support through telephone calls	n/a	• oxygen consumption (VO_2_ Max) significantly better in intervention group • no difference in number of exercise sessions or duration between groups	7
8	Lombard 1995 [18]	weekly or every 3 weeks	telephone	yes, counselor conducted a high structure prompt or low structure prompt	n/a	• groups that received weekly prompts walked significantly more than those prompted every 3 weeks (even after the intervention ended) • prompt structure had no significant effect on amount of walking	8
9	Marshall 2003 [19]	every 2 weeks	mail or email	yes, messages were tailored to stage of change	n/a	• no significant difference in amount of physical activity found between groups • participants inactive at baseline: both groups showed a positive change in total physical activity, but only significant for print group • decreased time sitting on a weekday observed for both groups, only significant for Web group • quarter of participants in both groups moved forward at least one stage	6
10	Napolitano 2003 [20]	weekly	email	none	n/a	• at one month, intervention group more likely to have moved forward in stage of change, had more moderate intensity minutes of exercise, and more walking minutes • at 3 months, only minutes spent walking remained significant between intervention and control groups	8
11	Petersen 2008 [21]	weekly	email	yes, individualized messages were sent to help participants stay on course	positive association found between use of intervention website and weight loss	• small, but statistically significant, positive changes in most dietary measures • higher percentage of participants in normal weight category compared to non-participants, but no difference in average weight change • increased website usage associated with more weight loss and stage of change improvement	5
12	Plotnikoff 2005 [22]	weekly	email	none	n/a	• intervention group found to be more active, have higher self-efficacy, perceive not being active as more of a threat to health, perceive more advantages and less disadvantages to being active, and have favorable changes in the dietary variables • effect sizes were small	9
13	Spittaels 2007 [23]	five messages over 8 weeks	email	yes, one group received messages tailored to stage of change	n/a	• all groups increased their activity levels, but no differences were found between groups • subgroup of participants who went through more thorough data collection: body fat significantly decreased in tailored plus email group • tailored advice reported to be remembered, printed out, and discussed more with others • more in tailored group reported to have changed their activity behavior after reading advice.	9
14	Svetkey 2008 [24]	weekly or monthly	email and/or telephone	yes, the personal contact group spoke or met with an interventionist monthly	n/a	• first 24 months: both the intervention groups gained significantly less weight than the control group • at 30 months: personal contact group regained significantly less weight than the other two groups • interactive technology-based group was not statistically different than control group at 30 months	9
15	Tate 2006 [25]	weekly	email	yes, study compared groups that received no feedback, automated tailored counseling, or feedback from a counselor	positive association found between use of free website (among control group) and number of diary submissions (intervention group) and weight loss	• at 3 months: two counseling groups did not differ from each other and had lost significantly more weight than website only group • at 6 months: human counseling group lost more weight than website only group, and automated counseling group not significantly different than the other two groups • greater use of free site associated with greater weight loss in the website only group • more diary submissions were associated with more weight loss in two counseling groups	8
16	Tate 2003 [26]	weekly	email	yes, counseling group received personalized feedback	n/a	• at 12 months: internet plus e-counseling group lost more weight than the internet only group	7
17	Tate 2001 [27]	weekly	email	yes, counseling group received personalized feedback	n/a	• behavioral therapy group lost more weight than Internet only group at three and 6 months	8
18	Williamson 2006 [28]	weekly	email	yes, counselor provided feedback on participant's progress with the program components	n/a	• at 6 months: interactive behavior therapy group lost more body fat than passive education group • at 24 months: no difference in weight between groups (the interactive group regained the lost weight)	8
19	Woodall 2007 [29]	every 5 weeks	email	none	positive association found between responding to reminder emails and positive dietary change	• participants were more likely to visit the site on the day a reminder e-mail was sent • responding to reminder emails associated with positive dietary change including increased fruit and vegetable intake	4

### Main Findings

Out of the 19 articles included in this review, 11 reported generally positive results regarding the use of periodic prompts (articles 1, 2, 3, 4, 5, 8, 12, 14, 15, 16, 17 in Table 2 and Table 3). This classification of study results as generally positive is based on whether periodic prompts themselves appeared to be supported, and not on whether the specific research questions the studies aimed to address were supported by the results.

The following section describes the articles’ main findings regarding prompt characteristics. Results from studies that compared specific aspects of interventions between groups are described in each section. First, we discuss the findings regarding prompt frequency and how weekly prompts compare with other periodicities. Then, to supplement that analysis and understand the conditions under which periodic prompts may be more or less effective, we examine medium used for prompt, intervention components, tailoring, and level of interaction with intervention. As appropriate, some studies are described more than once. Table 2 and Table 3 provide information on the research questions the studies were designed to answer and the main findings reported.

#### Frequency of Prompt

In examining the effectiveness of periodic prompts, the first question is what prompt frequency might be most effective. Only two studies specifically compared timing intervals for sending prompts. One intervention aimed at increasing walking sent prompts weekly to one group and every 3 weeks to another treatment group (article 8). Participants who were prompted every week walked for significantly more weeks than the participants who were prompted less often (based on survival analysis), and this statistically significant difference was maintained over a 3-month time frame post-intervention [18].

In the second study examining periodicity, a weight-loss maintenance intervention used weekly and monthly prompts with two different treatment groups, but it is difficult to draw conclusions regarding frequency of communication from this study because the weekly messages were automated emails and the monthly contact was with a weight gain prevention counselor mostly by telephone (article 14). At 24 months, the two intervention groups did not differ by weight regained, and the participants in the treatment groups regained significantly less weight than the no-treatment control group (data not available to calculate effect sizes) [24]. At 30 months, only the monthly personal contact group remained significantly better than the control group [24]. At 30 months the difference between the group that received weekly automated prompts and the control group was not statistically significant, and the difference between the two treatment groups was not statistically significant.

#### Medium Used for Prompt

The medium used to deliver periodic prompts may affect the outcome of a behavior change intervention. In a weight-loss intervention, the telephone group lost significantly more weight than the mail group at 6 months (0.12 kg difference [*P* < .01]), but at 12 months differences were not significant (article 6) [16].

The effect of a booklet with mailed reinforcements and a website with emailed messages on physical activity levels were compared (article 9). Both groups increased their activity and there was no significant difference in amount of physical activity between the groups [19]. Another study evaluated groups receiving telephone prompts, email prompts, and no prompts (article 14). One group received monthly personal contact mostly by telephone, the second intervention group had access to an interactive website and had to check in weekly, and the control group received no prompts [24]. The two interventions were significantly better at preventing weight regain than the control through 24 months of data collection (data necessary to calculate effect size not reported). At 30 months the personal contact group had regained less weight than the interactive website group (0.21 kg difference [*P* < .01]) and the control group (0.27 kg difference [*P* = .001]), and the interactive website group was not significantly different from the control group [24]. This result is hard to interpret, though, because the telephone group was receiving personal contacts and the email group’s prompts were automated.

#### Intervention Components

Most of the interventions described in the articles used multifaceted approaches to change participants’ behavior. Unfortunately, it is difficult to draw conclusions regarding effectiveness of prompts when additional components are used because no studies compared prompts alone to prompts with additional intervention tools.

Eight interventions included periodic prompts in association with online tools. Examples of tools used include quizzes, weight-tracking charts, goal setting, and bulletin boards. One study reported that participants with access to online tools, including periodic lessons and feedback, lost more weight than a group with access to only weight loss links at 3 months (0.84 kg difference [*P* = .001]) and 6 months (0.63 kg difference [*P* < .05]) (article 17) [27]. A similar study found that two groups assigned to a website with online tools, including periodic automated or counselor feedback, lost more weight at 3 months than a group assigned to a less comprehensive, free weight-loss site with periodic non-tailored email prompts (counseling group vs online tools only group 0.89 kg difference [*P* = .001] and automated feedback group vs online tools only group 0.65 kg difference [*P* < .01]) (article 15) [25]. The difference between the periodic automated feedback and non-tailored email prompt group was not significant at 6 months, and only the group with periodic feedback from a counselor in addition to online tools had better weight loss results than the email prompt control group at 6 months (0.79 kg difference [*P* < .001]) [25]. Finally, an intervention involving the extensive use of online tools and periodic personal feedback was compared to passive online education regarding weight loss (article 18). The periodic feedback and online tools group lost more body fat than the passive education group at 6 months (0.74 percent body fat difference [*P* < .05]), but the weight lost was gained back, and there was no significant difference between the groups at 24 months [28].

#### Tailoring

Health promotion researchers hypothesize that sending participants personalized prompts relevant to their own situation or interest is more effective at changing behavior than generic reminders, and have tested this idea [30]. This section focuses on the results of studies that tested the effects of tailoring prompts in conjunction with periodicity of prompts. Prompts were tailored by personal contact with a counselor or automated online information personalized using information provided by participants.

An intervention aimed at increasing physical activity tested the effect of highly structured prompts compared to non-structured prompts (article 8). High-structure prompts consisted of a research staff member providing specific feedback to the participant based on the walking behavior information they submitted the week prior, and the staff member and participant together setting a specific walking goal for the next week. Low-structure prompts consisted of a research staff member asking the participants how their walking was going. The final survival analysis showed that the structure of the prompt had no significant effect on walking behavior, and that all prompted groups increased their walking [18].

Monthly personal prompts, mostly by telephone, were compared to an online intervention with required weekly check ins and no tailoring (article 14). Both were aimed at preventing weight regain, and a control group was included. At 24 months, both treatment groups gained significantly less weight than the control group (data needed to calculate effect size not reported), but at 30 months the personal contact group had regained significantly less than the control group (0.27 kg difference [*P* < .01]) and the online group was not significantly different from the control group [24].

A weight-loss intervention compared three groups: one received no feedback and only had access to a basic website; one received automated, tailored periodic prompts and had access to a comprehensive website; and a third group received periodic feedback from a counselor and had access to the comprehensive website (article 15). At 3 months the two groups receiving feedback did not differ, and both had lost significantly more weight than the no feedback group (counseling group vs online tools only group 0.89 kg difference[*P* = .001]; automated feedback group vs online tools only group 0.65 kg difference [*P* < .01]) [25]. At 6 months, the counselor feedback group had lost significantly more weight than the no feedback group (0.79 kg difference [*P* < .001]), and the automated feedback group was not significantly different from either of the other two groups [25].

#### Level of Interaction with Intervention

Several studies reported differences in results in the frequency with which the participants responded to the periodic prompts or otherwise interacted with the intervention. Interaction can be measured by the number of emails opened, number of log ins to the intervention website, or number of weeks a participant remained in a program. Interpreting these findings is difficult because of the possibility that participants who were already motivated to change their behavior interacted with the intervention tools more than other participants.

In a nutrition intervention the number of weeks a participant interacted with the program was significantly related to forward progression in stage of change (article 1). Two weight loss studies that used periodic prompts reported that more weight loss was associated with more use of an intervention website (articles 5 and 11), which may have been increased because of prompting. In another study, greater use of a free website among a control group, and more diary submissions by two counseling groups, were found to be associated with greater weight loss (article 15). Finally, responding to periodic email prompts by visiting an intervention website was associated with positive dietary change in a nutrition intervention (article 19).

## Discussion

### Findings

As evidenced by 11 articles reporting generally positive effects of periodic prompts and 8 articles reporting mixed results, the evidence that periodic prompts can effectively enhance diet, weight loss, and exercise behaviors appears to be positive, but is not entirely consistent. The few studies that looked at prompt frequency did show that it affected intervention effectiveness, with one study in particular demonstrating that weekly prompts were significantly more useful than prompts given every 3 weeks (article 8). Questions remain regarding how prompts issued more than once a week, or even every day, would affect behavior change because these frequencies were not tested in any of the studies. The medium used to communicate prompts did not affect results (if personal feedback was not added to the intervention) in the two studies testing different media for delivering prompts; both found no differences in effectiveness (articles 6 and 9).

It is difficult to evaluate findings regarding the effectiveness of prompts within multi-component interventions because prompts, and other elements, were not often explicitly tested. In addition, components included in multi-component programs varied widely. Therefore, it is unknown if websites with more comprehensive sets of online tools are more effective at changing behavior than less comprehensive sites. When intervention components were implemented with one group and not another, often that was not the only difference between the groups. For example, if periodic prompts were also not communicated to the control group, then the effect of the intervention components was not tested. This makes it difficult to assess the value of individual intervention elements.

Tailoring periodic prompts through regular contact with a counselor produced positive results (articles 10, 16, 17, 19, 20, 23), especially when compared over time to groups not receiving personal contact (articles 14, 15, 17). Significant limitations regarding tailoring exist in the literature because often groups provided with personalized periodic prompts were compared to groups that were not given any prompts (articles 7, 17, 18). Contact with a counselor over the phone or by email is an important form of periodic prompting to consider because health behavior counselors can serve many more clients by using methods other than face-to-face contact. This finding poses a challenge, though, to those who are designing limited contact interventions to be automated for cost-effectiveness and other reasons. Comparing automated prompts to regular counselor contact is important because programs using automated prompts that are implemented to save resources need to know which prompt characteristics produce effects as close to those using prompts given by counselors. Cost benefit analyses would be beneficial for further understanding optimal program choices.

More interaction by participants with the periodic prompt intervention program was associated with better outcomes in several studies (articles 1, 5, 11, 15, 19). The association found between more interaction with intervention components and better results could be a reflection of self-selection among the most motivated participants, or it could be that certain people were more engaged because of the intervention itself, and that resulted in better outcomes, or both.

Long-term, sustainable behavior change and health benefits are not shown by this review because of the lack of follow-up data collection and results in the literature. Two of the six studies that incorporated follow-up data collection showed non-significant or inconclusive results (articles 11 and 18). In addition, the articles that did collect follow-up data used heterogeneous methods in terms of cessation of prompts, outcome measures, and time period following the intervention, and were therefore difficult to compare.

All of the studies reviewed, except one, had participants who were recruited and volunteered to participate in the interventions. It is possible that those who volunteer for health interventions are already motivated to change their behavior and are open to the information being sent to them. If this is true, then results of limited contact interventions with prompts implemented broadly may have less positive outcomes than the ones included in this review. From an alternative perspective, providing interventions with prompts to facilitate behavior change among those who are motivated to improve their health would be a valuable service.

Related to the self-selection issue is the non-equivalent participation by males and females in the studies reviewed. Women may be more likely to volunteer for these types of studies, as is illustrated by the high proportion of participants who were female in the combined sample of all the studies in this review. Little is known about how effective these interventions would be at changing the behaviors of men. Most studies did not report on differences in results among men in their sample, and the men who volunteer for these studies may produce different results than men in the general population.

Finally, to prove effectiveness of an emerging type of intervention, data must be collected to evaluate the results. Many of the studies included in this review required participants to visit study staff for the data to be collected. It is possible that behavior change may be partially attributed to the fact that participants know they will need to return to a study site for data collection. If this is the case, and this type of contact is not part of the program when implemented on a large scale, then the results of the intervention could be weaker than the results from the initial study.

### Limitations and Strengths of Studies

Multiple issues make it difficult to draw strong, generalizable conclusions about the effectiveness of limited contact interventions with prompts, including: (1) a lack of follow-up; (2) self-selected samples; (3) a higher proportion of female participants; (4) a lack of rigorous testing of intervention factors; and (5) data collection methods that might differ when an intervention is implemented on a broad scale. The body of literature on periodic prompt interventions also has strengths. It has produced relatively consistent results, which show promise for this type of behavior change program. Control groups, randomization, and follow-up data collection were used in several studies, and those findings were therefore strengthened. In addition, several of the studies had large sample sizes.

### Limitations and Strengths of Review

Despite using a thorough search strategy, there may be some literature on interventions using prompts that were not identified for this review. Specifically, we did not examine the grey literature (unpublished documents and reports) on this topic, focusing instead on data that had been through the peer-review process. A meta-analysis was not possible due to the various data collection methods and outcomes in the studies. Also, the variability in limited contact interventions regarding targeted behavior and methods utilized makes it difficult to develop generalized conclusions about their effectiveness.

Despite these limitations, this is the first literature review, to our knowledge, that examines the effectiveness of periodic prompts for changing diet, activity, and weight-loss behaviors. Insights regarding effectiveness of prompts and possible ways to make them more effective are presented in an organized manner, and future research directions in this area are recommended based on this review.

### Conclusions

In light of promising results from some studies, additional research on limited contact interventions is warranted. It would be valuable for future studies to use no-treatment control groups, include long-term follow-up data collection, and test specific intervention components or prompt characteristics instead of entire programs. In particular, further investigation into the effectiveness of different time intervals between prompts would be highly valuable. It would also be informative if researchers were able to include a more representative proportion of men in studies to see if they respond differently to these types of interventions.

New media has the potential to reach people in fresh and exciting ways. Examples of such media include text messages on cellular phones [31], and messages which could appear on social networking sites. Further research comparing reach and effectiveness of various types of communication technology is recommended.

This review shows that the use of periodic prompts can be effective in behavior change interventions. Effectiveness is enhanced if prompts are frequent and personal contact with a counselor is included. These findings can be used to improve interventions that use periodic prompts and will hopefully result in increased effectiveness, positive behavior change, and improved health.
